# The Canadian Network for Mood and Anxiety Treatments Task Force Recommendations for the Use of Probiotics, Prebiotics, Synbiotics, and Fecal Microbiota Transplants in Adults With Major Depressive Disorder: Recommandations du Groupe de travail du Réseau canadien pour le traitement des troubles de l’humeur et de l’anxiété (Canadian Network for Mood and Anxiety Treatments, CANMAT) concernant l’utilisation des probiotiques, des prébiotiques, des symbiotiques et de la transplantation de microbiote fécal chez les adultes atteints de trouble dépressif majeur

**DOI:** 10.1177/07067437251394363

**Published:** 2025-11-18

**Authors:** Anees Bahji, Elisa Brietzke, Noah C.A. Cooke, Fiona Clement, Benicio N. Frey, Mark Hofmeister, Sidney H. Kennedy, Raymond Lam, Roumen Milev, Dina Moinul, Sagar V. Parikh, Scott Patten, Arun Ravindran, Joshua D. Rosenblat, Zainab Samaan, Ayal Schaffer, April Saleem, Serge Beaulieu, Valérie Tourjman, Michael Van Ameringen, Simone Vigod, Lakshmi Yatham, Valerie Taylor, Canadian Network for Mood and Anxiety Treatments (CANMAT) Taskforce

**Affiliations:** 1 Department of Psychiatry, 3158 University of Calgary, Calgary, Canada; 2 Department of Community Health Sciences, University of Calgary, Calgary, Canada; 3 Department of Psychiatry, 4257 University of Toronto, Toronto, Canada; 4 Cumming School of Medicine, 12204 University of Calgary, Calgary, Canada; 5 MRC Epidemiology Unit, University of Cambridge, Cambridge, UK; 6 O’Brien Institute for Public Health, University of Calgary, Calgary, Canada; 7 Department of Psychiatry and Behavioural Neurosciences, 3710 McMaster University, Hamilton, Canada; 8 Health Technology Assessment Unit, University of Calgary, Calgary, Canada; 9 Department of Psychiatry, University of British Columbia, Vancouver, Canada; 10 Department of Psychiatry, 4257 Providence Care Hospital, Queen's University, Kingston, Canada; 11 Department of Psychiatry, 1259 University of Michigan, Ann Arbor, USA; 12 Department of Pathology and Molecular Medicine, 4257 Queen's University, Kingston, Canada; 13 Department of Psychiatry, McGill University, Montreal, Canada; 14 Department of Psychiatry, 5622 Université de Montréal, Montreal, Canada; 15 Women's College Hospital, Toronto, Ontario, Toronto, Canada

**Keywords:** major depressive disorder, gut–brain axis, probiotics, fecal microbiota transplantation, microbiome-based therapies, adjunctive treatments, CANMAT guidelines, psychobiotics, mental health interventions, systematic review

## Abstract

**Background:**

Approximately one-third of adults with major depressive disorder (MDD) experience limited response or intolerable side effects with existing pharmacotherapies. As such, innovative treatments targeting novel biological pathways are under investigation. One promising area of research is the gut microbiome and its influence on mood through the microbiota–gut–brain axis. Clinical studies have begun evaluating microbiome-targeted interventions such as probiotics, prebiotics, synbiotics, and fecal microbiota transplantation (FMT) as potential treatments for MDD. The Canadian Network for Mood and Anxiety Treatments (CANMAT) convened a task force to evaluate the evidence for microbiome-targeted interventions in adults with MDD and to provide updated clinical recommendations.

**Methods:**

A systematic review of randomized controlled trials (RCTs) and meta-analyses was conducted, assessing interventions such as probiotics, prebiotics, synbiotics, and FMT in adults with MDD. The CANMAT methodology was used to determine levels of evidence and treatment line recommendations, which were presented in a question-and-answer format.

**Results:**

Twenty-three RCTs and eight meta-analyses were included. Probiotics have been the most extensively studied and have demonstrated modest improvements in depressive symptoms, particularly when used in an adjunctive manner. However, recent high-quality trials yielded mixed results. Evidence for prebiotics and FMT was limited and inconclusive, while synbiotics were assessed in only one small RCT. Most interventions were well tolerated, with few serious adverse events.

**Conclusions:**

Probiotics may be cautiously considered as third-line adjunctive treatments for MDD, though findings remain inconsistent. There is currently insufficient evidence to recommend prebiotics, synbiotics, or FMT in clinical practice. Further large-scale, well-controlled trials are needed to clarify efficacy, safety, and optimal patient subgroups.

## Résumé

**Contexte:**

Environ un tiers des adultes atteints d’un trouble dépressif majeur (TDM) présentent une réponse limitée ou des effets secondaires intolérables avec les pharmacothérapies existantes. C’est pourquoi des traitements novateurs ciblant de nouvelles voies biologiques sont à l’étude. Un domaine de recherche prometteur est celui du microbiome intestinal et de son influence sur l’humeur par le biais de l’axe microbiote-intestin-cerveau. Des études cliniques ont commencé à évaluer des interventions ciblant le microbiome comme les probiotiques, les prébiotiques, les symbiotiques et la transplantation de microbiote fécal (TMF) comme traitements potentiels du TDM. Le Réseau canadien pour le traitement des troubles de l’humeur et de l’anxiété (CANMAT) a mis sur pied un groupe de travail chargé d’évaluer les données probantes sur les interventions ciblant le microbiome chez les adultes atteints de TDM et de formuler des recommandations cliniques à jour.

**Méthodes:**

On a procédé à une revue systématique des essais contrôlés à répartition aléatoire (ECRA) et des méta-analyses, évaluant les interventions comme les probiotiques, les prébiotiques, les symbiotiques et la TMF chez les adultes atteints de TDM. La méthodologie de CANMAT a été utilisée pour déterminer les niveaux de preuves et les recommandations thérapeutiques, qui ont été présentées sous forme de questions et réponses.

**Résultats:**

Vingt-trois ECRA et huit méta-analyses ont été inclus. Les probiotiques ont fait l’objet des études les plus poussées et ont démontré une amélioration modeste des symptômes dépressifs, en particulier lorsqu’ils sont utilisés en complément. Cependant, des essais récents de grande qualité ont donné des résultats mitigés. Les données probantes concernant les prébiotiques et la TMF étaient limitées et non concluantes, tandis que les symbiotiques n’ont été évalués que dans un seul petit ECRA. La plupart des interventions ont été bien tolérées, avec peu d’effets indésirables graves.

**Conclusions:**

Les probiotiques peuvent être envisagés avec prudence comme traitement d’appoint de troisième intention pour le TDM, bien que les résultats restent incohérents. Il n’existe actuellement pas suffisamment de preuves pour recommander les prébiotiques, les symbiotiques ou la TMF dans la pratique clinique. D’autres essais à grande échelle et bien contrôlés sont nécessaires pour clarifier l’efficacité, l’innocuité et les sous-groupes de patients optimaux.

## Introduction

The human microbiota, comprising approximately 100 trillion microorganisms, plays a vital role in maintaining homeostasis and regulating a wide range of physiological processes.^1^ Most of these microbes reside in the gastrointestinal tract, where they interact intimately with the host's immune, endocrine, and nervous systems. The bidirectional communication between the gut and brain—commonly referred to as the microbiota–gut–brain axis—is increasingly recognized as a key factor in mental health.^2^ This axis integrates neural, hormonal, and immune pathways, linking gut function to mood regulation, cognition, and emotional processing.

Disruptions in the gut microbiome have been associated with psychiatric symptoms, particularly in individuals with comorbid gastrointestinal conditions such as inflammatory bowel disease.^3–5^ An expanding body of research suggests that alterations in microbial diversity and composition may contribute to the pathophysiology of major depressive disorder (MDD) through mechanisms such as increased gut permeability, chronic low-grade inflammation, and impaired neurotransmitter synthesis.

In response, microbiome-targeted interventions have emerged as potential novel treatments for MDD. These include probiotics (live microorganisms), prebiotics (nondigestible compounds that promote beneficial bacteria), synbiotics (combinations of probiotics and prebiotics), para-probiotics (inactivated microbial components—also referred to in the literature as postbiotics or parabiotics), and fecal microbiota transplantation (FMT)—are being studied as novel strategies to modulate this axis and potentially improve mood symptoms.^2,6^ These interventions may act through a range of mechanisms, including modulation of neurotransmitter systems (e.g., serotonin, γ-aminobutyric acid), regulation of immune responses, enhancement of gut barrier integrity, reduction of systemic inflammation, and normalization of hypothalamic–pituitary–adrenal axis activity.^7–10^

Although preclinical data and early clinical trials suggest that modulating the gut microbiome may influence depressive symptoms, findings have been mixed. Variability in study design, population characteristics, microbial strains, and outcome measures has contributed to inconsistent results.^11,12^ For example, a recent meta-analysis of 34 RCTs concluded that microbiome-targeted interventions are modestly effective in reducing depressive symptoms overall (SMD −0.26), but their efficacy may vary depending on geography, comorbidity status, and treatment duration.^13^ This variability underscores the need for clinically grounded, consensus-based guidance to aid in interpretation and implementation. As a result, clinical guidance on the use of these interventions remains limited.

To address this gap, the Canadian Network for Mood and Anxiety Treatments (CANMAT) convened a multidisciplinary task force to critically evaluate the role of microbiome-targeted interventions in the treatment of MDD. This Task Force Report presents the results of a systematic review of randomized controlled trials (RCTs) and meta-analyses, synthesizing the available evidence to inform clinical recommendations rather than pooled effect sizes. Although recent meta-analyses, such as those by Pan et al. (2025), have quantified the overall antidepressant effects of microbiome-targeted therapies, they do not translate this evidence into practice-oriented guidance. In contrast, the CANMAT report uses structured consensus methods—consistent with its prior guideline development process—to bridge evidence with clinical applicability. In addition to summarizing efficacy, safety, and tolerability, the report highlights key limitations in the current literature and outlines priorities for future research, policy development, and implementation. This report does not aim to duplicate existing statistical meta-analyses, but rather to contextualize and apply them to guide real-world treatment decisions.

## Methods

### Scope and Structure

This review followed a modified version of the methodology used in prior CANMAT guidelines.^14–16^ A systematic review was conducted in accordance with the 2020 Preferred Reporting Items for Systematic Reviews and Meta-Analyses (PRISMA) guidelines.^17^ Although a protocol was developed internally to guide the search and selection processes, it was not formally registered due to evolving project scope and timelines. Future reviews will prioritize prospective protocol registration to enhance transparency and accountability.

As with prior CANMAT guidelines, clinical applicability was prioritized by organizing the review around six key questions identified through structured consensus among the task force. These questions focused on efficacy, safety, and tolerability of microbiome interventions as monotherapy or augmentation strategies in various clinical populations. The resulting framework informed both the review structure and the final recommendations.

### Eligibility Criteria

We included RCTs and systematic reviews of RCTs involving adults (≥18 years) with MDD, where participants received microbiome-targeted interventions (probiotics, prebiotics, synbiotics, para-probiotics, or FMT). Studies were required to report validated mental health outcomes, including depressive or anxiety symptom ratings. Studies were excluded if they:

- Focused solely on biological or hormonal endpoints without clinical outcomes.
- Included populations with primary substance-use disorders.
- Were not peer-reviewed (e.g., abstracts, posters).
- Lacked full-text availability or were narrative reviews.

Inclusion criteria were refined in consultation with clinical experts and a research librarian to ensure face validity and optimal search sensitivity.

### Information Sources and Search Strategy

The following databases were searched from inception to 28 February 2025: MEDLINE, Embase, PsycINFO, the Cochrane Database of Systematic Reviews, and the Cochrane Central Register of Controlled Trials. The search strategy combined terms related to microbiome-targeted interventions (e.g., probiotics, prebiotics, FMT) with those related to mood disorders. A health sciences librarian developed and peer-reviewed the strategy using the PRESS (Peer Review of Electronic Search Strategies) checklist.^18^ Reference lists of included studies and relevant guidelines were also manually screened.

### Selection Process

Titles and abstracts were independently screened by two reviewers (DM, AS) following a calibration exercise. Full texts were reviewed in duplicate, with discrepancies resolved by consensus. Inclusion was determined conservatively—any study marked as relevant by either reviewer was carried forward for full-text review.

### Data Collection and Items Extracted

Data extraction was conducted independently in duplicate using standardized forms. Extracted items included author, year, study design, setting, sample size, intervention details (type, strain, dose), duration, participant demographics, mental health outcomes, and adverse events.

### Risk of Bias Assessment

This report represents a CANMAT Task Force guideline and therefore combines features of systematic and narrative reviews. While we conducted a structured search and applied explicit inclusion/exclusion criteria, our primary aim was to synthesize the evidence into practice-oriented recommendations rather than to produce pooled effect sizes. Consistent with previous CANMAT guidelines, we did not apply formal quality assessment tools (e.g., Cochrane RoB 2.0, AMSTAR-2, or GRADE) to our review. This decision reflects the heterogeneity and emerging nature of the evidence base as well as the precedent established in prior CANMAT Task Force reports. Instead, we qualitatively considered study design features, sample size, outcome measures, and population characteristics when interpreting results. We acknowledge this approach as a limitation but believe it supports the pragmatic goal of generating consensus-based recommendations in an evolving field.

### Synthesis Methods

Due to heterogeneity in populations, interventions, and outcome measures, meta-analysis was not feasible. Instead, the findings were synthesized narratively in a question-and-answer format, consistent with previous CANMAT reports.^19–22^ Studies were grouped by intervention type and analyzed for consistency, effect size, and risk of bias. Certain studies may be discussed under multiple questions if they addressed more than one clinical context (e.g., adjunctive and monotherapy use), in line with CANMAT's question-driven structure.

### Appraisal of Evidence and Recommendation Ratings

Clinical recommendations were developed using CANMAT's adapted grading framework, which integrates both the strength of empirical evidence and its clinical applicability. Levels of evidence (Table 1) ranged from Level 1 (meta-analyses or replicated large RCTs) to Level 4 (pilot studies or expert-informed rationale). Treatment lines (Table 2) ranged from first-line to “not recommended,” based on a combination of efficacy, safety, tolerability, and real-world feasibility. In instances where the literature was sparse, conflicting, or highly heterogeneous, structured expert consensus was used to interpret findings within a clinical context. This approach is consistent with prior CANMAT Task Force recommendations and aligns with international best practices for guideline development in emerging fields. While formal levels of evidence provide a useful hierarchy, we acknowledge their limitations and emphasize that clinical judgment remains essential when applying research findings to individualized care. Broader system-level factors such as cost, accessibility, and patient acceptability were also considered for higher-order recommendations.

**Table 1.** Definitions for Level of Evidence Ratings.

Level	Evidence criteria
1	Meta-analyses with narrow confidence intervals, or replicated double-blind randomized controlled trials (RCTs) with adequate sample size (*n* ≥ 30 per arm).
2	Meta-analyses with wide confidence intervals, or a single well-powered double-blind RCT (*n* ≥ 30 per arm).
3	At least one double-blind RCT with smaller samples (*n* = 10–29 per arm), or high-quality health system administrative data.
4	Pilot studies (*n* < 10 per arm), uncontrolled trials, anecdotal evidence, or expert opinion.

**Table 2.** Definitions for Line of Treatment Ratings.

Line of treatment	Evidence level criteria
First-line	Supported by Level 1 or Level 2 evidence and endorsed by clinical consensus on safety, tolerability, and feasibility.
Second-line	Supported by Level 3 or higher evidence with task force agreement on clinical appropriateness.
Third-line	Supported by Level 4 evidence or by expert opinion in the absence of more robust data.
Not recommended	Supported by Level 1 or 2 evidence indicating a lack of efficacy, with consensus agreement.

Where relevant, qualitative descriptors of treatment effect size (e.g., “modest”) were informed by estimates reported in existing meta-analyses. For example, a standardized mean difference (SMD) of approximately 0.2–0.3 has been described as a modest effect size in prior CANMAT reports and relevant literature. However, as this review did not conduct a formal meta-analysis, such descriptors are used narratively and not as formal thresholds.

## Results

### Overview

A total of 4,825 records were identified through the systematic search. After title and abstract screening, 102 full-text articles were reviewed. Ultimately, 23 RCTs and eight meta-analyses met the inclusion criteria. These studies formed the basis for addressing the six clinical questions developed by the CANMAT Task Force. Probiotics were the most extensively studied intervention, while evidence for prebiotics, synbiotics, and FMT was limited or preliminary. Several meta-analyses included overlapping trials, and the included studies exhibited substantial heterogeneity in intervention strains, sample characteristics, and outcome measures.

### Heterogeneity Across Trials

The microbiome literature is characterized by considerable heterogeneity, which precluded formal meta-analysis in this review. Across the 23 included RCTs and eight meta-analyses, there was substantial variability in:

- **Intervention type and formulation**: Studies investigated different probiotic strains (e.g., *Lactobacillus*, *Bifidobacterium*, *Clostridium butyricum*), combinations (multispecies vs. single strain), or delivery formats (capsules, fermented foods).
- **Dosage and duration**: Probiotic doses ranged widely (from 10^8^ to 10¹¹ CFU/day), and treatment durations varied from 2 to 12 weeks.
- **Treatment strategy**: Some trials evaluated microbiome-targeted therapies as adjunctive treatments, while others investigated them as monotherapy, particularly in antidepressant-naïve populations.
- **Population characteristics**: Clinical samples varied in terms of baseline depression severity, presence of comorbidities (e.g., gastrointestinal conditions, metabolic syndrome), and geographical region, which is known to influence gut microbial composition.
- **Outcome measurement tools**: Depressive symptoms were assessed using a range of validated scales, including the Hamilton Depression Rating Scale (HAM-D), PHQ-9, and MADRS, which limited the comparability of effect sizes across trials.

These factors likely contribute to the inconsistencies observed in trial-level findings and in prior meta-analyses. As such, this guideline chose to summarize findings narratively, in alignment with CANMAT's established consensus-based methodology, and to prioritize clinical applicability over statistical pooling.

## Question 1: Do Probiotics, Prebiotics, Synbiotics, and FMT Improve Depressive Symptoms in Adults With MDD?

**Figure 1.**
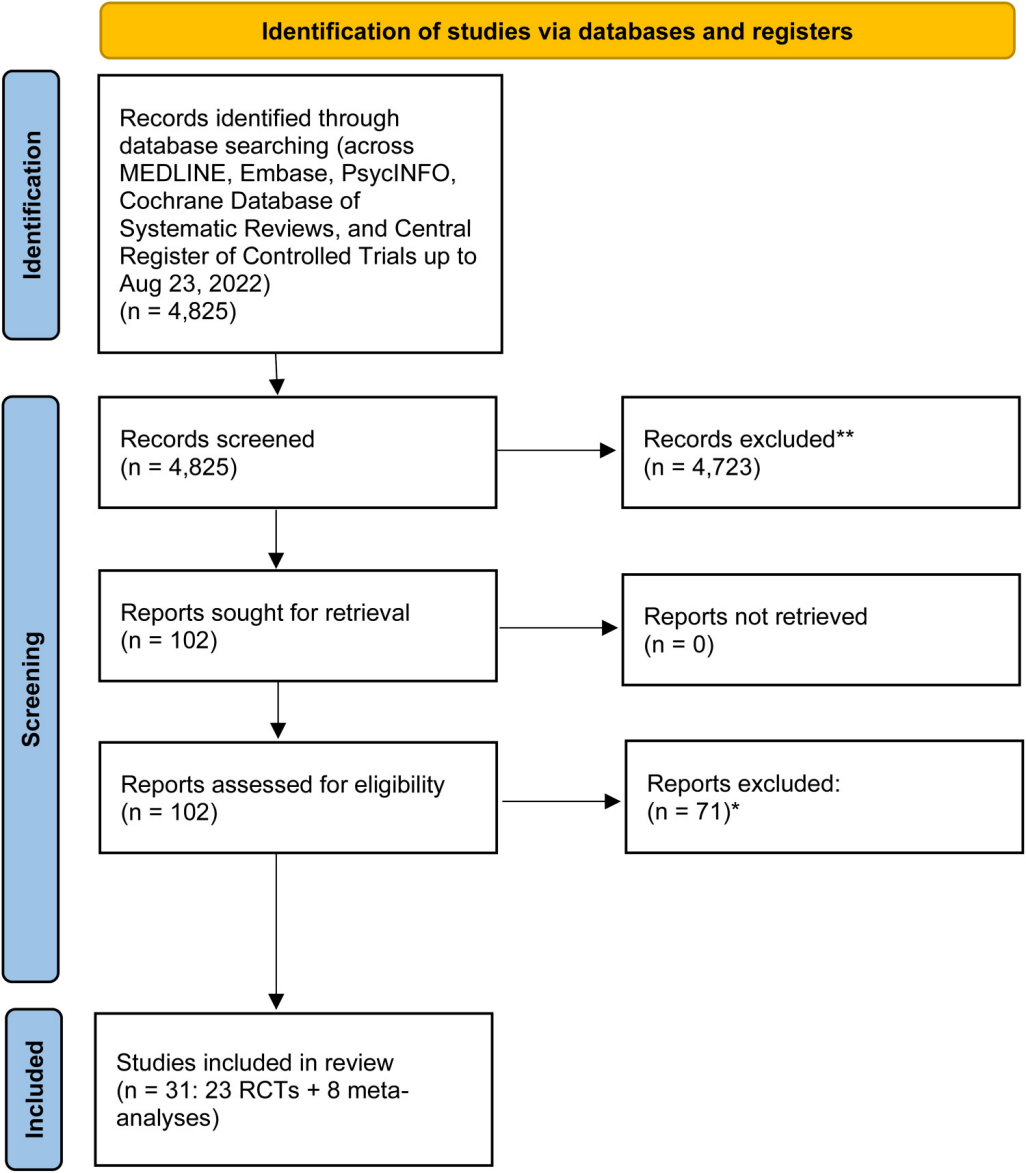
PRISMA flow diagram—study selection.

*Note.* HAM D = Hamilton Depression Rating Scale. * Of the 71 excluded full-text articles, reasons included ineligible study design (e.g., observational or animal studies), wrong population, insufficient outcome data, or not focused on MDD. *Note.* The initial systematic search and screening process was conducted by two independent reviewers (DM and AS), with calibration and expert consultation as noted in the original protocol. Records were retrieved across six databases with broad search terms related to probiotics and mood outcomes. The PRISMA diagram reflects both the original search and the refined updates undertaken during manuscript development. Due to project evolution, some screening steps and duplicate exclusions were not formally tracked and are not displayed here. PRISMA = Preferred Reporting Items for Systematic Reviews and Meta-Analyses.

A growing body of evidence from both meta-analyses and RCTs has examined the antidepressant potential of microbiome-targeted therapies, particularly probiotics. Our analysis of the RCTs in Table 3 reveals that 12 studies (*N* = 781) reported statistically significant symptom improvements. In comparison, 11 studies (*N* = 795) did not. Although no formal meta-analysis was conducted for this report, this “box score” approach provides a practical summary of the current evidence base (Figure 1).

**Table 3.** Summary of Randomized Controlled Trials Evaluating Microbiome-Targeted Interventions in Adults With Major Depressive Disorder (MDD).

Study	Country/population	Intervention	Duration	Primary outcome	Main findings
Akkasheh et al. 2016^31^	Iran, DSM-IV MDD (moderate-severe), *N* = 40 (37y, 85% female)	MS probiotic + SSRI	8 weeks	BDI	Significant improvement
Arifdjanova et al. 2021^48^	Russia, ICD-10 MDD (mild-moderate), *N* = 119 (33y, 62% female)	MS probiotic + SSRI	6 weeks	HAMD-17	No significant change
Baião et al. 2022^32^	UK, DSM-IV MDD (mild-moderate), *N* = 71 (29y, 63% female)	MS probiotic (standalone)	4 weeks	PHQ-9	Significant improvement
Chahwan et al. 2019^49^	Australia, BDI-II ≥ 12, *N* = 71 (36y, 69% female)	Multi-strain probiotic (standalone)	8 weeks	BDI	No significant change
Gawlik-Kotelnicka et al. 2023^50^	Poland, MDD, *N* = 60 (35y, 85% female)	Duo-strain probiotic	60 days	MADRS	No significant change
Gawlik-Kotelnicka et al. 2024^44^	Poland, DSM-5 MDD, *N* = 116 (34y, 15% female)	Duo-strain probiotic	60 days	MADRS	NS overall; significant clinical response in subgroup
Ghorbani et al. 2018^35^	Iran, DSM-5 MDD (moderate), *N* = 40 (35y, 70% female)	Synbiotic + SSRI	6 weeks	HAMD-17	Significant improvement
Green et al. 2023^36^	Australia, DSM-5 MDD (moderate-severe), *N* = 15 (44y, 60% female)	FMT (enema)	8 weeks	MADRS	No significant change
Hashemi-Mohammadabad et al. 2024^51^	Iran, HDRS > 20 MDD (moderate), *N* = 112 (35y, 100% female)	MS probiotics + SSRIs	8 weeks	HDRS	Significant improvement
Huang et al. 2019^52^	China, ICD-10 MDD (severe), *N* = 104 (age not reported)	Bifidobacterium spp. + electroacupuncture	8 weeks	HAMD-17	Significant improvement
Kazemi et al. 2019^30^	Iran, ICD-10 MDD (mild-moderate), *N* = 74 (36y, 71% female)	Duo-strain probiotic + SSRI	8 weeks	BDI	Significant for probiotic, NS for prebiotic
Lin et al. 2024^34^	Taiwan, DSM-5 MDD (moderate), *N* = 32 (38y, 75% female)	*Lactobacillus* *plantarum* PS128 + antidepressant	8 weeks	HAMD-17	No significant change
Majeed et al. 2018^53^	Iran, ICD-10 MDD (mild-moderate), *N* = 40 (42y, 85% female)	*Bacillus* *coagulans* + antidepressant	90 days	HAMD-17	Significant improvement
Nikolova et al. 2023^29^	UK, DSM-5 MDD (moderate), *N* = 49 (32y, 80% female)	MS probiotic + antidepressant	8 weeks	HAMD-17	Significant improvement
Reininghaus et al. 2020^54^	Austria, ICD-10 MDD (severe), *N* = 61 (42y, 77% female)	MS probiotic + biotin	4 weeks	BDI-II	No significant change
Romijin et al. 2017^41^	New Zealand, QIDS-SR at least moderate, *N* = 79 (35y, 79% female)	Duo-strain probiotic	8 weeks	MADRS	No significant change
Rudzki et al. 2019^55^	Poland, DSM-IV-TR MDD (moderate), *N* = 60 (39y, 71% female)	*L*. *plantarum* + SSRI	8 weeks	HAMD-17	No significant change
Saccarello et al. 2020^56^	Italy, ICD-10 MDD (mild-moderate), *N* = 90 (48y, 82% female)	SAMe + *L. plantarum*	6 weeks	Z-SDS	Significant improvement
Schaub et al. 2022^33^	Switzerland, ICD-10 MDD (moderate-severe), *N* = 47 (39y, 57% female)	MS probiotic + TAU	31 days	HAMD-17	Significant improvement
Strodl et al. 2024^42^	Australia, DSM-5 MDD (mild-to-moderate), *N* = 120 (38y, 69% female)	MS probiotic + CoQ10 + Mg	8 weeks	HAMD-17	Improved at 4 weeks, NS at 8 and 16 weeks
Tian et al. 2022^37^	China, ICD-10 MDD (mild-moderate), *N* = 45 (50y, 50% female)	B. breve + SSRI/SNRI	4 weeks	HAMD-17	Significant improvement
Vaghef-Mehrabany et al. 2021^57^	Iran, ICD-10 MDD (mild-moderate), *N* = 62 (39y, 100% female)	Prebiotic + SSRI/SNRI	8 weeks	HDRS	No significant change
Zhang et al. 2021^39^	China, ICD-10 MDD (mild-moderate), *N* = 69 (48y, 64% female)	*Lacticaseibacillus* *paracasei* Shirota	9 weeks	HAMD-17	Significant improvement

Overall, findings suggest modest symptom reductions, especially when probiotics are used as adjuncts to antidepressants. However, results remain heterogeneous and appear to vary depending on study design, intervention type, and population characteristics.

Several meta-analyses support the efficacy of probiotics in adults with MDD. Amirani et al. (2020) reported a weighted mean difference of −9.60 on the HAM-D, along with reductions in CRP, IL-10, and malondialdehyde levels, suggesting anti-inflammatory and antioxidative effects.^23^ Chao et al. (2020) found an SMD of −0.47 in depressive symptoms, especially in individuals with clinical mood disorders or under high stress.^24^ Goh et al. (2019) synthesized data from 19 RCTs (*n* = 1901) and reported a small but significant effect (SMD = –0.24) for probiotics, with stronger results in clinically diagnosed MDD samples.^12^ Other meta-analyses have similarly reported small but consistent effects, especially in adults under 60 years of age.^25,26^

A 2025 meta-analysis by Pan et al. further supports these findings, reporting a small yet significant pooled effect (SMD = –0.26) for microbiome-targeted treatments overall.^13^ Notably, this benefit was more pronounced in studies from Asia and in participants without gastrointestinal comorbidities. At the same time, no significant effects were observed in perinatal depression or with treatment durations exceeding 12 weeks.

Not all meta-analyses have found uniform benefits. Ng et al. (2018) reported no significant main effect; however, subgroup analyses suggested a benefit for mild-to-moderate depression.^27^ Nikolova et al. (2019) initially reported a very large effect size (SMD = 1.371), but a 2021 update revised this to a more conservative and credible estimate (SMD = 0.83), particularly for adjunctive use.^11,28^ Differences in methodology, sample characteristics, and probiotic formulations likely explain these discrepancies.

RCT-level findings are similarly mixed but generally encouraging. In a double-blind RCT, Nikolova et al. (2023) found that probiotic augmentation of SSRIs significantly reduced HAM-D and Hamilton Anxiety Rating Scale (HAM-A) scores in partial responders.^29^ Kazemi et al.^30^ and Akkasheh et al.^31^ each found that eight weeks of probiotic supplementation significantly improved depressive symptoms compared to placebo, with Akkasheh also reporting improvements in metabolic markers. Baiao et al. (2023) observed improvements in PHQ-9 scores and emotional processing after four weeks of multispecies probiotics.^32^ Schaub et al. (2022) demonstrated symptom reduction and changes in brain activity with high-dose probiotic supplementation.^33^ In contrast, Lin et al. (2024) reported no significant differences between probiotic and placebo groups despite changes in gut microbiota composition.^34^

Evidence for prebiotics and synbiotics remains limited. A small trial by Ghorbani et al. (2018) found that synbiotics combined with fluoxetine resulted in greater symptom reductions compared to a placebo.^35^ However, prebiotics alone have not demonstrated significant antidepressant effects in MDD.

FMT remains in an early investigational stage. A pilot RCT by Green et al. (2023) found that FMT was feasible and well tolerated in adults with moderate-to-severe MDD, but the study was underpowered to evaluate efficacy.^36^ Most other studies of FMT are either preclinical or observational in nature.

In summary, probiotics appear to offer modest antidepressant benefits, particularly when used as adjunctive therapies in adults with MDD. Evidence for prebiotics, synbiotics, and FMT remains sparse and preliminary.

## Question 2: Are These Interventions Effective as Adjunctive Treatments in Adults Already Taking Antidepressants?

A growing number of randomized trials have explored whether microbiome-based interventions can enhance the effects of antidepressant medications in individuals with partial or incomplete responses. This section evaluates the evidence for their efficacy as adjunctive treatments.

Adjunctive probiotic therapy has received the most empirical support to date. Several well-conducted RCTs demonstrate that when probiotics are combined with antidepressants, they may lead to greater symptom reductions than antidepressants alone. For instance, in partial responders to SSRIs, Nikolova et al. (2023) reported statistically and clinically significant decreases in both depression and anxiety scores.^29^ Tian et al. (2022) showed that adjunctive *Bifidobacterium breve* not only improved depressive symptoms but also regulated serotonin turnover and gastrointestinal symptoms, highlighting a potential gut–brain interaction mechanism.^37^

Schaub et al. (2022) found that high-dose probiotics maintained microbial diversity and reduced depressive symptoms in individuals on pharmacotherapy.^33^ Similarly, Majeed et al. (2018) and Zhang et al. (2021) documented improvements in mood and comorbid gastrointestinal symptoms.^38,39^ Meta-analyses by Goh et al. (2019) and Nikolova et al. (2021) both conclude that probiotics are most beneficial as adjuncts rather than standalone therapies.^11,12^

However, not all adjunctive trials have been positive. Lin et al. (2024) reported no significant group differences, despite improvements in both arms.^34^ Variability in strain selection, treatment duration, and population characteristics (e.g., degree of treatment resistance or metabolic comorbidity) may influence outcomes.

There is insufficient evidence to support the use of prebiotics or synbiotics as adjunctive treatments. Although Ghorbani et al. (2018) reported an enhanced fluoxetine response with synbiotics, no replication studies have been conducted.^35^ No RCTs have assessed FMT in combination with antidepressants.

In conclusion, probiotics may be considered as third-line adjunctive options in adults with partial response to antidepressants. The utility of other interventions in this context remains unproven.

## Question 3: Are These Interventions Effective as Monotherapy in Antidepressant-Naïve Individuals?

Given interest in non-pharmacologic alternatives, some studies have assessed microbiome-targeted therapies as standalone interventions in antidepressant-naïve individuals. This section reviews their potential role as monotherapy in the treatment of MDD.

Probiotics have been examined as standalone treatments in a small number of trials involving antidepressant-naïve individuals with MDD. Baiao et al. (2023) observed a reduction in depressive symptoms and an enhancement in emotional processing following four weeks of multispecies probiotics.^32^ Otaka et al. (2021) and Zhang et al. (2021) reported improvements in mood, constipation, and inflammatory markers in patients not on concurrent antidepressant therapy.^39,40^

However, other studies have yielded null or equivocal findings. Romijn et al. (2017), which included individuals with subthreshold depressive symptoms, found no difference between probiotics and placebo.^41^ Strodl et al. (2024) evaluated a probiotic supplement combined with magnesium and CoQ10 in adults with MDD, reporting early improvement that did not persist beyond four weeks, raising questions about the durability of effects and the specific contribution of probiotics.^42^

There are currently no RCTs of prebiotics, synbiotics, or FMT used as monotherapy in MDD. Available studies in these domains primarily focus on the adjunctive use of these treatments in populations without a formal diagnosis of MDD.

Overall, the evidence base for probiotics as monotherapy in antidepressant-naïve adults is limited and inconsistent. These interventions cannot currently be recommended as standalone treatments for MDD.

## Question 4: Are These Interventions Effective in Individuals With Treatment-Resistant Depression?

Treatment-resistant depression (TRD) represents a severe and often refractory clinical presentation. Although limited in number, some trials have begun to assess whether microbiome-based interventions can provide benefit in this difficult-to-treat population.

Miyaoka et al. (2018) conducted an open-label trial of *Clostridium butyricum* (CBM588) in individuals with TRD and reported significant symptom improvement.^43^ This strain may exert beneficial effects through short-chain fatty acid production, but controlled trials are lacking.

Nikolova et al. (2023) included participants with inadequate response to antidepressants and found that probiotic supplementation led to further improvements in mood and anxiety.^29^ However, the sample was not limited to TRD, and subgroup results were exploratory.

No studies have examined the efficacy of prebiotics, synbiotics, or FMT in defined TRD populations. Given the limited evidence, probiotics may have a role as adjunctive options in TRD, but stronger data from large RCTs are required before any formal recommendations can be made.

## Question 5: Do These Interventions Work Better in Specific Subgroups?

Individual patient factors may influence treatment response to microbiome-targeted interventions. This section explores subgroup analyses and emerging predictors of differential efficacy.

Emerging evidence suggests that the efficacy of microbiome-targeted interventions may differ across patient subgroups. Gawlik-Kotelnicka et al. (2024) found that participants with higher levels of baseline stress experienced greater reductions in depressive symptoms with probiotics.^44^ Conversely, individuals with metabolic conditions (e.g., obesity, central adiposity) demonstrated reduced treatment responsiveness, possibly due to chronic inflammation or altered microbiota composition.

Age may also be an important moderator. Meta-analyses indicate that probiotics may be more effective in individuals under 60 years old, possibly due to the preservation of gut microbial diversity and more intact epithelial signalling. Older adults may exhibit diminished mucosal response to probiotics, although direct mechanistic studies are sparse.

Another potential moderator is baseline microbiome composition. While few trials prospectively stratify by microbiota profile, evidence from both animal and human studies suggests that individuals with depleted microbial diversity or low abundance of specific strains (e.g., *Lactobacillus*, *Bifidobacterium*) may derive greater benefit from supplementation.^9,45^

These findings remain exploratory, and future RCTs should incorporate stratified designs to validate predictors of response. Until then, clinicians may consider a patient's age, stress level, and metabolic profile when deciding on the use of probiotics.

## Question 6: Are These Interventions Safe and Well-Tolerated in Adults With MDD?

Safety and tolerability are key considerations for any adjunctive or alternative therapy. This section summarizes reported adverse events and overall tolerability of microbiome-based interventions in adults with MDD.

Overall, probiotics, prebiotics, and synbiotics appear to be safe and well-tolerated in adult populations with MDD. Most trials report only mild gastrointestinal side effects such as gas, bloating, and transient discomfort. Serious adverse events are rare, though isolated cases of bacteraemia have been reported in critically ill or immunocompromised patients receiving probiotic therapy.^46,47^

Prebiotics and synbiotics have shown favourable safety profiles in small trials, with no consistent adverse effects. However, long-term data are lacking, and tolerability in older adults or those with comorbid conditions remains underexplored. FMT, as reported in Green et al. (2023), was feasible and well-tolerated in MDD participants, with no serious adverse events; however, the intervention remains investigational and subject to regulatory scrutiny.^36^

An additional concern is the incomplete reporting of safety outcomes across studies. Meta-reviews have highlighted a consistent lack of adverse event detail in probiotic trials, which limits firm conclusions on safety.^47^ Para-probiotics (non-viable bacterial products), also referred to as postbiotics, are an emerging class of microbiome-based interventions. While preclinical studies suggest potential mood-related benefits, these effects may differ from those of live probiotics, and human safety data remain limited. Further research is required to understand their clinical relevance and safety profile.

In sum, microbiome-targeted therapies have generally been well tolerated in clinical trials involving medically stable adults with MDD. However, there is limited concrete evidence on long-term safety, strain-specific risks, and optimal dosing. Adverse events are inconsistently reported, and the absence of harm cannot be assumed to indicate safety. In addition, cost, accessibility, and feasibility were not systematically assessed and warrant further consideration. Caution remains essential, particularly in high-risk populations, and more rigorous safety monitoring is needed.

## Limitations

This report was developed through a task force process and reflects a structured narrative review rather than a formal systematic review or meta-analysis. Although we followed PRISMA-aligned principles where feasible, including a comprehensive literature search and predefined inclusion criteria, we did not conduct independent dual screening, formal risk of bias assessment, or protocol registration. As such, our synthesis may be subject to selection and reporting bias.

We did not systematically assess the quality of included RCTs or meta-analyses using validated tools such as the Cochrane Risk of Bias 2.0 or AMSTAR-2. Instead, study findings were interpreted qualitatively, with attention to sample size, intervention fidelity, and outcome measurement.

The probiotic evidence base is limited by substantial heterogeneity in intervention characteristics. Most trials used different bacterial strains, variable dosages, and treatment durations ranging from four to 12 weeks. Furthermore, many studies examined probiotics as adjunctive therapies, while others evaluated them as monotherapy, making it difficult to isolate their specific effects. This variability complicates direct comparisons and limits our ability to issue strain-specific or dose-dependent recommendations.

Safety outcomes were inconsistently reported across studies, and few trials evaluated long-term tolerability or risk in high-risk populations. Additionally, cost, accessibility, and product variability were not addressed in most trials and were beyond the scope of this review.

In addition, heterogeneity across trials—particularly in probiotic strains, dosing, treatment durations, and patient populations—limits the generalizability of our conclusions. Data on long-term safety, cost-effectiveness, and accessibility remain sparse, and most studies excluded medically complex or high-risk populations. These gaps underscore the need for cautious interpretation and further high-quality research to guide clinical practice.

## Conclusions

This CANMAT Task Force review synthesizes current evidence on microbiome-targeted interventions for adults with MDD. Probiotics show modest benefit as a third-line adjunctive treatment, particularly in those with partial response to antidepressants, and are generally well tolerated. While this classification reflects the presence of multiple double-blind RCTs (Level 3 evidence), the Task Force downgraded probiotics to third-line due to several factors: lack of consistent replication across specific strains, substantial heterogeneity in formulation, dose, and patient characteristics, and practical challenges related to product standardization, regulatory oversight, and real-world implementation. These limitations reduce the generalizability and clinical applicability of current findings. Routine clinical use of probiotics cannot yet be recommended outside of individualized adjunctive treatment. Evidence for prebiotics and FMT remains insufficient, and no recommendations can be made for synbiotics due to a lack of data from RCTs.

Among the microbiome-targeted interventions reviewed, probiotics have been the most frequently studied and have demonstrated some preliminary signals of clinical benefit, particularly when used as adjunctive treatments. However, findings across trials remain mixed and limited by significant methodological variability, including differences in strain, dose, treatment duration, and patient population. Although a few studies suggest that certain subgroups—such as individuals with elevated stress levels or specific microbial profiles—may experience greater benefit, these hypotheses remain exploratory and untested in prospectively designed trials. FMT remains a promising but investigational approach, and its use should be restricted to controlled research settings. Currently, the evidence base for prebiotics and synbiotics is limited, and no clinical recommendations can be made for their use.

Importantly, microbiome-targeted interventions appear to be safe and well tolerated in medically stable adults with MDD, with adverse events that are typically mild and gastrointestinal in nature. However, safety reporting remains inconsistent, and caution is warranted in patients who are immunocompromised or have medical complexities.

Future research should prioritize well-powered, rigorously controlled trials in clinical populations, with a focus on diagnostic clarity, standardized outcome measurement, and subgroup stratification. Mechanistic studies integrating microbiome, immunologic, and metabolomic data may help elucidate pathways of response. The standardization of probiotic formulations and eventual reclassification of some products as live biotherapeutics could also enhance regulatory oversight and therapeutic development.

While microbiome-based treatments represent a promising frontier in depression care, they remain investigational. Their use in clinical practice should be individualized, cautiously applied, and firmly grounded in evidence-informed care principles.

## Supplemental Material

sj-docx-1-cpa-10.1177_07067437251394363 - Supplemental material for The Canadian Network for Mood and Anxiety Treatments Task Force Recommendations for the Use of Probiotics, Prebiotics, Synbiotics, and Fecal Microbiota Transplants in Adults With Major Depressive Disorder: Recommandations du Groupe de travail du Réseau canadien pour le traitement des troubles de l’humeur et de l’anxiété (Canadian Network for Mood and Anxiety Treatments, CANMAT) concernant l’utilisation des probiotiques, des prébiotiques, des symbiotiques et de la transplantation de microbiote fécal chez les adultes atteints de trouble dépressif majeur Supplemental material, sj-docx-1-cpa-10.1177_07067437251394363 for The Canadian Network for Mood and Anxiety Treatments Task Force Recommendations for the Use of Probiotics, Prebiotics, Synbiotics, and Fecal Microbiota Transplants in Adults With Major Depressive Disorder: Recommandations du Groupe de travail du Réseau canadien pour le traitement des troubles de l’humeur et de l’anxiété (Canadian Network for Mood and Anxiety Treatments, CANMAT) concernant l’utilisation des probiotiques, des prébiotiques, des symbiotiques et de la transplantation de microbiote fécal chez les adultes atteints de trouble dépressif majeur by Anees Bahji, Elisa Brietzke, Noah C.A. Cooke, Fiona Clement, Benicio N. Frey, Mark Hofmeister, Sidney H. Kennedy, Raymond Lam, Roumen Milev, Dina Moinul, Sagar V. Parikh, Scott Patten, Arun Ravindran, Joshua D. Rosenblat, Zainab Samaan, Ayal Schaffer, April Saleem, Serge Beaulieu, Valérie Tourjman, Michael Van Ameringen, Simone Vigod, Lakshmi Yatham, Valerie Taylor and in The Canadian Journal of Psychiatry

sj-docx-2-cpa-10.1177_07067437251394363 - Supplemental material for The Canadian Network for Mood and Anxiety Treatments Task Force Recommendations for the Use of Probiotics, Prebiotics, Synbiotics, and Fecal Microbiota Transplants in Adults With Major Depressive Disorder: Recommandations du Groupe de travail du Réseau canadien pour le traitement des troubles de l’humeur et de l’anxiété (Canadian Network for Mood and Anxiety Treatments, CANMAT) concernant l’utilisation des probiotiques, des prébiotiques, des symbiotiques et de la transplantation de microbiote fécal chez les adultes atteints de trouble dépressif majeur Supplemental material, sj-docx-2-cpa-10.1177_07067437251394363 for The Canadian Network for Mood and Anxiety Treatments Task Force Recommendations for the Use of Probiotics, Prebiotics, Synbiotics, and Fecal Microbiota Transplants in Adults With Major Depressive Disorder: Recommandations du Groupe de travail du Réseau canadien pour le traitement des troubles de l’humeur et de l’anxiété (Canadian Network for Mood and Anxiety Treatments, CANMAT) concernant l’utilisation des probiotiques, des prébiotiques, des symbiotiques et de la transplantation de microbiote fécal chez les adultes atteints de trouble dépressif majeur by Anees Bahji, Elisa Brietzke, Noah C.A. Cooke, Fiona Clement, Benicio N. Frey, Mark Hofmeister, Sidney H. Kennedy, Raymond Lam, Roumen Milev, Dina Moinul, Sagar V. Parikh, Scott Patten, Arun Ravindran, Joshua D. Rosenblat, Zainab Samaan, Ayal Schaffer, April Saleem, Serge Beaulieu, Valérie Tourjman, Michael Van Ameringen, Simone Vigod, Lakshmi Yatham, Valerie Taylor and in The Canadian Journal of Psychiatry

sj-docx-3-cpa-10.1177_07067437251394363 - Supplemental material for The Canadian Network for Mood and Anxiety Treatments Task Force Recommendations for the Use of Probiotics, Prebiotics, Synbiotics, and Fecal Microbiota Transplants in Adults With Major Depressive Disorder: Recommandations du Groupe de travail du Réseau canadien pour le traitement des troubles de l’humeur et de l’anxiété (Canadian Network for Mood and Anxiety Treatments, CANMAT) concernant l’utilisation des probiotiques, des prébiotiques, des symbiotiques et de la transplantation de microbiote fécal chez les adultes atteints de trouble dépressif majeur Supplemental material, sj-docx-3-cpa-10.1177_07067437251394363 for The Canadian Network for Mood and Anxiety Treatments Task Force Recommendations for the Use of Probiotics, Prebiotics, Synbiotics, and Fecal Microbiota Transplants in Adults With Major Depressive Disorder: Recommandations du Groupe de travail du Réseau canadien pour le traitement des troubles de l’humeur et de l’anxiété (Canadian Network for Mood and Anxiety Treatments, CANMAT) concernant l’utilisation des probiotiques, des prébiotiques, des symbiotiques et de la transplantation de microbiote fécal chez les adultes atteints de trouble dépressif majeur by Anees Bahji, Elisa Brietzke, Noah C.A. Cooke, Fiona Clement, Benicio N. Frey, Mark Hofmeister, Sidney H. Kennedy, Raymond Lam, Roumen Milev, Dina Moinul, Sagar V. Parikh, Scott Patten, Arun Ravindran, Joshua D. Rosenblat, Zainab Samaan, Ayal Schaffer, April Saleem, Serge Beaulieu, Valérie Tourjman, Michael Van Ameringen, Simone Vigod, Lakshmi Yatham, Valerie Taylor and in The Canadian Journal of Psychiatry
