# Supplementary material for: The Canadian Network for Mood and Anxiety Treatments Task Force Recommendations for the Use of Probiotics, Prebiotics, Synbiotics, and Fecal Microbiota Transplants in Adults With Major Depressive Disorder: Recommandations du Groupe de travail du Réseau canadien pour le traitement des troubles de l’humeur et de l’anxiété (Canadian Network for Mood and Anxiety Treatments, CANMAT) concernant l’utilisation des probiotiques, des prébiotiques, des symbiotiques et de la transplantation de microbiote fécal chez les adultes atteints de trouble dépressif majeur
Source: Can J Psychiatry. 2025 Nov 18:07067437251394363. Online ahead of print. doi: 10.1177/07067437251394363 (PMC12626857; doi:10.1177/07067437251394363)
Supplement: sj-docx-1-cpa-10.1177_07067437251394363 - Supplemental material for The Canadian Network for Mood and Anxiety Treatments Task Force Recommendations for the Use of Probiotics, Prebiotics, Synbiotics, and Fecal Microbiota Transplants in Adults With Major Depressive Disorder: Recommandations du Groupe  [file sj-docx-1-cpa-10.1177_07067437251394363.docx]

**Preferred Reporting Items for Systematic reviews and Meta-Analyses extension for Scoping Reviews (PRISMA-ScR) Checklist**

| **SECTION** | **ITEM** | **PRISMA-ScR CHECKLIST ITEM** | **REPORTED ON PAGE #** |
| --- | --- | --- | --- |
| **TITLE** | | | |
| Title | 1 | Identify the report as a scoping review. | Page 1 |
| **ABSTRACT** | | | |
| Structured summary | 2 | Provide a structured summary that includes (as applicable): background, objectives, eligibility criteria, sources of evidence, charting methods, results, and conclusions that relate to the review questions and objectives. | Page 2 (Abstract provides a structured summary of background, objectives, methods, results, and conclusions) |
| **INTRODUCTION** | | | |
| Rationale | 3 | Describe the rationale for the review in the context of what is already known. Explain why the review questions/objectives lend themselves to a scoping review approach. | Page 3 (Introduction outlines the rationale for exploring microbiome-targeted interventions for MDD) |
| Objectives | 4 | Provide an explicit statement of the questions and objectives being addressed with reference to their key elements (e.g., population or participants, concepts, and context) or other relevant key elements used to conceptualize the review questions and/or objectives. | Page 3 |
| **METHODS** | | | |
| Protocol and registration | 5 | Indicate whether a review protocol exists; state if and where it can be accessed (e.g., a Web address); and if available, provide registration information, including the registration number. | Page 4 (Methods indicate a protocol was developed internally but not registered). |
| Eligibility criteria | 6 | Specify characteristics of the sources of evidence used as eligibility criteria (e.g., years considered, language, and publication status), and provide a rationale. | Page 4 |
| Information sources* | 7 | Describe all information sources in the search (e.g., databases with dates of coverage and contact with authors to identify additional sources), as well as the date the most recent search was executed. | Page 4 (Information Sources and Search Strategy: MEDLINE, Embase, PsycINFO, Cockrane databases and search date February 28, 2025) |
| Search | 8 | Present the full electronic search strategy for at least 1 database, including any limits used, such that it could be repeated. | Page 4 (Methods describe the search strategy combining terms for microbiome interventions and mood disorders; detail available from authors) |
| Selection of sources of evidence† | 9 | State the process for selecting sources of evidence (i.e., screening and eligibility) included in the scoping review. | Page 4 (Selection Process in Methods describe independent title/abstract and full-text screening by two reviewers with consensus resolution) |
| Data charting process‡ | 10 | Describe the methods of charting data from the included sources of evidence (e.g., calibrated forms or forms that have been tested by the team before their use, and whether data charting was done independently or in duplicate) and any processes for obtaining and confirming data from investigators. | Pages 4-5 |
| Data items | 11 | List and define all variables for which data were sought and any assumptions and simplifications made. | Page 4 (Data Collection and Items Extracted: author, year, study design, sample size, intervention type/strain/dose, duration, participant demographics outcomes, adverse events) |
| Critical appraisal of individual sources of evidence§ | 12 | If done, provide a rationale for conducting a critical appraisal of included sources of evidence; describe the methods used and how this information was used in any data synthesis (if appropriate). | Not applicable (This task force report followed a narrative review methodology aligned with prior CANMAT guidelines. No formal critical appraisal or risk-of-bias assessment conducted) |
| Synthesis of results | 13 | Describe the methods of handling and summarizing the data that were charted. | Page 5 (Narrative synthesis grouped by intervention type due to heterogeneity) |
| **RESULTS** | | | |
| Selection of sources of evidence | 14 | Give numbers of sources of evidence screened, assessed for eligibility, and included in the review, with reasons for exclusions at each stage, ideally using a flow diagram. | Page 5 (Results: 4,825 title and abstracts screened, 102 full-text articles screened; 23 RCTs and 8 meta-analyses included. Reasons for exclusion summarized) |
| Characteristics of sources of evidence | 15 | For each source of evidence, present characteristics for which data were charted and provide the citations. | Pages 5-6 (Characteristics of included studies summarized; Table 3 details individual RCTs) |
| Critical appraisal within sources of evidence | 16 | If done, present data on critical appraisal of included sources of evidence (see item 12). | Not applicable (No formal critical appraisal within sources of evidence) |
| Results of individual sources of evidence | 17 | For each included source of evidence, present the relevant data that were charted that relate to the review questions and objectives. | Pages 5-8 (Results describe findings of individual RCTs and meta-analyses in relation to review questions) |
| Synthesis of results | 18 | Summarize and/or present the charting results as they relate to the review questions and objectives. | Pages 5-9 (Narrative summary of outcomes across interventions addressing each question) |
| **DISCUSSION** | | | |
| Summary of evidence | 19 | Summarize the main results (including an overview of concepts, themes, and types of evidence available), link to the review questions and objectives, and consider the relevance to key groups. | Pages 6-10 (Summarizes main findings: modest benefits for probiotics as adjunctive therapy) |
| Limitations | 20 | Discuss the limitations of the scoping review process. | Page 9-10 (Discussion acknowledges limitations including heterogeneity, lack of protocol registration, and absence of formal risk-of-bias assessment) |
| Conclusions | 21 | Provide a general interpretation of the results with respect to the review questions and objectives, as well as potential implications and/or next steps. | Page 2 and 5-9 (Abstract and Results) |
| **FUNDING** | | | |
| Funding | 22 | Describe sources of funding for the included sources of evidence, as well as sources of funding for the scoping review. Describe the role of the funders of the scoping review. | NO funding was obtained for this review. Not reported Funding sources. |

JBI = Joanna Briggs Institute; PRISMA-ScR = Preferred Reporting Items for Systematic reviews and Meta-Analyses extension for Scoping Reviews.

* Where *sources of evidence* (see second footnote) are compiled from, such as bibliographic databases, social media platforms, and Web sites.

† A more inclusive/heterogeneous term used to account for the different types of evidence or data sources (e.g., quantitative and/or qualitative research, expert opinion, and policy documents) that may be eligible in a scoping review as opposed to only studies. This is not to be confused with *information sources* (see first footnote).

‡ The frameworks by Arksey and O’Malley (6) and Levac and colleagues (7) and the JBI guidance (4, 5) refer to the process of data extraction in a scoping review as data charting*.*

§ The process of systematically examining research evidence to assess its validity, results, and relevance before using it to inform a decision. This term is used for items 12 and 19 instead of "risk of bias" (which is more applicable to systematic reviews of interventions) to include and acknowledge the various sources of evidence that may be used in a scoping review (e.g., quantitative and/or qualitative research, expert opinion, and policy document).

*From:* Tricco AC, Lillie E, Zarin W, O'Brien KK, Colquhoun H, Levac D, et al. PRISMA Extension for Scoping Reviews (PRISMAScR): Checklist and Explanation. Ann Intern Med. 2018;169:467–473. [doi: 10.7326/M18-0850](http://annals.org/aim/fullarticle/2700389/prisma-extension-scoping-reviews-prisma-scr-checklist-explanation).
