# Supplementary material for: The Canadian Network for Mood and Anxiety Treatments Task Force Recommendations for the Use of Probiotics, Prebiotics, Synbiotics, and Fecal Microbiota Transplants in Adults With Major Depressive Disorder: Recommandations du Groupe de travail du Réseau canadien pour le traitement des troubles de l’humeur et de l’anxiété (Canadian Network for Mood and Anxiety Treatments, CANMAT) concernant l’utilisation des probiotiques, des prébiotiques, des symbiotiques et de la transplantation de microbiote fécal chez les adultes atteints de trouble dépressif majeur
Source: Can J Psychiatry. 2025 Nov 18:07067437251394363. Online ahead of print. doi: 10.1177/07067437251394363 (PMC12626857; doi:10.1177/07067437251394363)
Supplement: sj-docx-2-cpa-10.1177_07067437251394363 - Supplemental material for The Canadian Network for Mood and Anxiety Treatments Task Force Recommendations for the Use of Probiotics, Prebiotics, Synbiotics, and Fecal Microbiota Transplants in Adults With Major Depressive Disorder: Recommandations du Groupe  [file sj-docx-2-cpa-10.1177_07067437251394363.docx]

**Appendix 1. Search Protocol and Strategy Summary**

**Objective:**

To identify randomized controlled trials (RCTs) and meta-analyses evaluating microbiome-targeted interventions (i.e., probiotics, prebiotics, synbiotics, para-probiotics, or fecal microbiota transplantation [FMT]) for the treatment of major depressive disorder (MDD) in adults.

**Eligibility Criteria:**

- **Population:** Adults (≥18 years) diagnosed with major depressive disorder (MDD) using validated clinical criteria (e.g., DSM-IV, DSM-5, ICD-10).
- **Intervention:** Any microbiome-targeted therapy, including probiotics, prebiotics, synbiotics, para-probiotics, or FMT.
- **Comparator:** Placebo or standard care.
- **Outcomes:** Depressive symptoms assessed using validated clinical rating scales (e.g., HAM-D, MADRS, PHQ-9).
- **Study Type:** RCTs or systematic reviews/meta-analyses of RCTs.
- **Exclusions:** Studies without clinical outcomes, non-peer-reviewed materials, abstracts only, case reports, or those focusing primarily on other diagnoses (e.g., substance use disorders).

**Search Strategy:**

The search strategy was developed by the lead author and peer-reviewed by a health sciences librarian. Searches were conducted across three timepoints using the following databases: PubMed, Embase, PsycINFO, Cochrane Database of Systematic Reviews, and Cochrane Central Register of Controlled Trials.

**Search Terms Used (PubMed example):**

(Probiotics OR Prebiotics OR Synbiotics OR Para-probiotics OR Lactobacilli OR Bifidobacteria OR "Fecal microbiota transplant" OR FMT) AND (Mood OR Anxiety OR Depression)

**Search Limits Applied:**

- Date: August 23, 2022 – February 28, 2025
- Language: English or French
- Study Type: Randomized controlled trials
- Population: Adults (≥19 years)
- Exclusions: Preprints, non-human studies

**Database Searched: PubMed**

Search String:

(Probiotics OR Prebiotics OR Synbiotics OR Para-probiotics OR Lactobacilli OR Bifidobacteria OR "Fecal microbiota transplant" OR FMT) AND (Mood OR Anxiety OR Depression)

**Search Terms (applied to PubMed):**

(Probiotics OR Prebiotics OR Synbiotics OR Para-probiotics OR Lactobacilli OR Bifidobacteria OR "Fecal microbiota transplant" OR FMT) AND (Mood OR Anxiety OR Depression)

**Appendix 1, Table 1: Search strategy**

| **Search Date** | **Step** | **Filters Applied** | **Records Retrieved** | **Records Included** |
| --- | --- | --- | --- | --- |
| April 8, 2024 | 1 | No filters | 5,454 | – |
| 2 | Publication date: Aug 23, 2022 – Dec 12, 3000 | 1,248 | – |  |
| 3 | Randomized Controlled Trial | 69 | – |  |
| 4 | Adults (19+), English or French, exclude preprints | 21 | 4 |  |
| February 28, 2025 | 1 | No filters | 6,731 | – |
| 2 | Publication date: Aug 23, 2022 – Dec 12, 3000 | 838 | – |  |
| 3 | Randomized Controlled Trial | 54 | – |  |
| 4 | Adults (19+), English or French, exclude preprints | 50 | 3 |  |
| April 6, 2023 | 1 | No filters | 4,825 | – |
| 2 | Title/abstract screened for eligibility | – | – |  |
| 3 | Full-text reviewed | 102 | – |  |
| 4 | Full-text excluded | 42 | – |  |
| 5 | Final studies included | – | 23 RCTs + 8 MAs |  |

**Included Studies**

- **RCTs (n = 23):** Included trials examined probiotics, prebiotics, synbiotics, or FMT in adults with MDD, with a focus on depressive symptom outcomes.
- **Meta-Analyses (n = 8):** Synthesized pooled data on microbiome-targeted interventions, primarily involving probiotics.

**Protocol Registration:**

This review followed an internal protocol developed by the lead author and team to guide screening and extraction processes. While not formally registered, the protocol is available upon request and aligned with PRISMA 2020 guidelines.
