# Supplementary material for: The Canadian Network for Mood and Anxiety Treatments Task Force Recommendations for the Use of Probiotics, Prebiotics, Synbiotics, and Fecal Microbiota Transplants in Adults With Major Depressive Disorder: Recommandations du Groupe de travail du Réseau canadien pour le traitement des troubles de l’humeur et de l’anxiété (Canadian Network for Mood and Anxiety Treatments, CANMAT) concernant l’utilisation des probiotiques, des prébiotiques, des symbiotiques et de la transplantation de microbiote fécal chez les adultes atteints de trouble dépressif majeur
Source: Can J Psychiatry. 2025 Nov 18:07067437251394363. Online ahead of print. doi: 10.1177/07067437251394363 (PMC12626857; doi:10.1177/07067437251394363)
Supplement: sj-docx-3-cpa-10.1177_07067437251394363 - Supplemental material for The Canadian Network for Mood and Anxiety Treatments Task Force Recommendations for the Use of Probiotics, Prebiotics, Synbiotics, and Fecal Microbiota Transplants in Adults With Major Depressive Disorder: Recommandations du Groupe  [file sj-docx-3-cpa-10.1177_07067437251394363.docx]

**Appendix 2.** Characteristics of Microbiome-Based Interventions in Included RCTs

| Study | Category | Strain(s) | Frequency | Design |
| --- | --- | --- | --- | --- |
| Akkasheh et al. 2016^31^ | MS probiotic | L. acidophilus (2x10^9), L. casei (2x10^9), B. bifidum (2x10^9) | One capsule daily | Add-on to SSRI (citalopram) |
| Arifdjanova et al. 2021^48^ | MS probiotic | L. acidophilus, L. rhamnosus, L. casei, L. bulgaricus, B. bifidum, B. longum, S. thermophilus (each at 2x10^9 cfu/g) | One capsule three times a day | Add-on to SSRI (escitalopram) |
| Baião et al. 2022^32^ | MS probiotic | 14-species blend including Bacillus, Bifidobacterium, Lactobacillus, Lactococcus, and Streptococcus strains (each at 2x10^9 cfu/g) | One capsule daily | Standalone |
| Chahwan et al. 2019^49^ | MS probiotic | B. bifidum, B. lactis, L. acidophilus, L. brevis, L. casei, L. salivarius, L. lactis (2.5x10^9) | 4 g daily | Standalone |
| Gawlik-Kotelnicka et al. 2023^50^ | Duo-strain probiotic | L. helveticus, B. longum | One capsule daily | Standalone |
| Gawlik-Kotelnicka et al. 2024^44^ | Duo-strain probiotic | L. helveticus, B. longum (each at 2x10^9 cfu/g) | One capsule daily | Standalone |
| Ghorbani et al. 2018^35^ | Synbiotic + SSRI | L. casaei, L. acidophilus, L. bulgaricus, L. rhamnosus, B. breve, B. longum, S. thermophilus + prebiotic (each at 2x10^9 cfu/g) | One capsule daily | Add-on to SSRI (fluoxetine) |
| Green et al. 2023^36^ | FMT (enema) | Enema-delivered FMT vs. placebo | One enema | Standalone |
| Hashemi-Mohammadabad et al. 2024^51^ | MS probiotics + SSRIs | L. acidophilus, B. bifidus, L. rutri, L. fermentum (each at 2x10^9 cfu/g) | One capsule daily | Add-on to SSRI |
| Huang et al. 2019^52^ | Single-strain probiotic | Bifidobacterium spp (at 2x10^9 cfu/g) | 1.5 grams, 3 times per day | Add-on to electroacupuncture |
| Kazemi et al. 2019^30^ | Duo-strain probiotic | L. helveticus, B. longum (each at 2x10^9) | 5 g daily | Add-on to SSRI or amitriptyline |
| Lin et al. 2024^34^ | Single-strain probiotic | L. plantarum PS128 (at 2x10^9 cfu/g) | One capsule daily | Add-on to SSRI |
| Majeed et al. 2018^53^ | B. coagulans + antidepressant | B. coagulans (2x10^9 CFU) | Two doses per day | Add-on to antidepressant |
| Nikolova et al. 2023^29^ | MS probiotic + antidepressant | 14-strain probiotic (Bacillus, Bifidobacterium, Lactobacillus, Lactococcus, Streptococcus; each at 2x10^9 cfu/g) | Four capsules daily | Add-on to antidepressant |
| Reininghaus et al. 2020^54^ | MS probiotic + biotin | B. bifidum, B. lactis, L. acidophilus, L. casei, L. paracasei, L. plantarum, L. salivarius, L. lactis (each at 2x10^9 cfu/g) | One capsule daily | Add-on to antidepressant |
| Romijin et al. 2017^41^ | Duo-strain probiotic | L. helveticus, B. longum (each at 2x10^9 cfu/g) | 1.5 g daily | Standalone |
| Rudzki et al. 2019^55^ | Single-strain probiotic | L. plantarum (at 1x10^9) | Two capsules daily | Add-on to SSRI |
| Saccarello et al. 2020^56^ | Single-strain probiotic | SAMe 200 mg + L. plantarum (at 1x10^9) | One capsule daily | Add-on to SAM-e |
| Schaub et al. 2022^33^ | MS probiotic | S. thermophilus, B. breve, B. lactis, L. acidophilus, L. plantarum, L. paracasei, L. delbrueckii, L. helveticus | 900 billion CFU/day | Add-on to TAU |
| Strodl et al. 2024^42^ | MS probiotic | L. acidophilus, B. bifidum, S. thermophilus | One capsule daily | Add-on to CoQ10 and Magnesium |
| Tian et al. 2022^37^ | Single-strain probiotic | B. breve (at 2x10^9 cfu/g)) | One capsule daily | Add-on to SSRI/SNRI |
| Vaghef-Mehrabany et al. 2021^57^ | Prebiotic | Inulin | 10 g/day | Add-on to SSRI/SNRI |
| Zhang et al. 2021^39^ | Single-strain probiotic | L. paracasei shirota (1x10^8 CFU) | One beverage/day | Add-on to SSRI/SNRI |
